# Size Dependence of the Tetragonal to Orthorhombic Phase Transition of Ammonia Borane in Nanoconfinement

**DOI:** 10.3390/ma17225672

**Published:** 2024-11-20

**Authors:** Shah Najiba, Jiuhua Chen, Mohammad S. Islam, Yongzhou Sun, Andriy Durygin, Vadym Drozd

**Affiliations:** 1 Center for the Study of Matter at Extreme Conditions, Department of Mechanical and Materials Engineering, Florida International University, Miami, FL 33199, USA; najiba.buet@gmail.com (S.N.);; 2 Department of Electrical Engineering and Computer Science, Case Western Reserve University, Cleveland, OH 44106, USA

**Keywords:** ammonia borane, nanoconfinement, phase transition, low temperature, in situ Raman spectroscopy, mesoporous silica scaffold

## Abstract

We have investigated the thermodynamic property modification of ammonia borane via nanoconfinement. Two different mesoporous silica scaffolds, SBA-15 and MCM-41, were used to confine ammonia borane. Using in situ Raman spectroscopy, we examined how pore size influences the phase transition temperature from tetragonal (*I4mm*) to orthorhombic (*Pmn21*) for ammonia borane. In bulk ammonia borane, the phase transition occurs at around 217 K; however, confinement in SBA-15 (with ~8 nm pore sizes) reduces this temperature to approximately 195 K, while confinement in MCM-41 (with pore sizes of 2.1–2.7 nm) further lowers it to below 90 K. This suppression of the phase transition as a function of pore size has not been previously studied using Raman spectroscopy. The stability of the *I4mm* phase at a much lower temperature can be interpreted by incorporating the surface energy terms to the overall free energy of the system in a simple thermodynamic model, which leads to a significant increase in the surface energy when transitioning from the tetragonal phase to the orthorhombic phase.

## 1. Introduction

The nanoconfinement of ammonia borane (NH_3_BH_3_) inside periodic mesoporous silica leads to a remarkable enhancement of its dehydrogenation kinetics [1]. When grain size decreases down to nanometers, surface energy becomes significant with regard to the internal energy of the system; therefore, the phase transition temperature/pressure may be altered depending on the relative surface energy across the transition. The size dependence of transition pressure has been reported in many studies [2,3,4,5,6]. The discovery of mesoporous silica with ordered uniform pore sizes, MCM-41 (Mobil Composition of Matter No. 41), early in 1992 by Mobil Corporation [7], followed by SBA-15 (Santa Baraba Amorphous-15), with larger ordered uniform pore sizes [8], in 1998 has stimulated a wide variety of research including fundamental studies of phase transitions in confined spaces, the effect of pore size and surface characteristics on ion exchange, and the inclusion of various metal complexes and other guests [9,10,11,12].

Ammonia borane (AB) has attracted significant research attention as a high-potential hydrogen storage material due to its high gravimetric and volumetric hydrogen densities [13]. Recent research interests in AB have focused on chemical modification [14,15,16,17,18], acid catalysis [19,20], and application of high pressure [21,22,23,24,25,26,27,28,29], etc., to improve the hydrogen storage property of AB. It has been reported that the nanoconfinement of ammonia borane inside a periodic mesoporous silica scaffold shows dramatically improved hydrogen storage properties in many aspects: nanoconfinement significantly improves the kinetics of hydrogen discharge at lower temperatures and reduces the contamination of unexpected toxic byproducts (e.g., borazine, etc.) [1,30,31]. Even though the improvement is striking, the mechanism responsible for the enhanced properties is not known, in part because of the absence of insight into the structural changes in AB inside mesoporous silica. We have conducted a comparative study of a temperature-induced tetragonal to orthorhombic phase transition in molecular crystal ammonia borane using nanoconfinements with different pore sizes, thus controlling the grain size of ammonia borane.

At ambient conditions, bulk ammonia borane crystallizes as a body-centered tetragonal (*I4mm*) structure [32,33], which transforms to the low temperature orthorhombic (*Pmn2*_1_) phase at ~218–225 K during cooling [25,26,34,35,36] with a significant change in the lattice dynamics, although the molecular structure of NH_3_BH_3_ molecule is preserved. In the *I4mm* phase, the molecular axes are aligned along the lattice c-axis, while the molecular axes become inclined relative to the c-axis in the orthorhombic phase. Kim et al. [37] used a atomic pair distribution function analysis of synchrotron X-ray powder diffraction data to follow the structural evolution of AB within MCM-41 at temperatures ranging from 80 K to 300 K. They found that the nanophase AB residing within the mesoporous scaffolds stays in the tetragonal phase at a much lower temperature than the neat AB molecular crystal and suggested that the nanoconfinement of AB within the mesoporous scaffolds stabilizes the AB structure at a lower temperature. Paolone et al. [38] used anelastic spectroscopy and differential scanning calorimetry (DSC) to investigate the structural phase transition of AB dispersed in the MCM-41 nanoscaffold and reported results similar to those reported by Kim et al. In our study, we utilized two different mesoporous silica scaffolds, SBA-15 and MCM-41, to systematically investigate how pore size influences the transition from the tetragonal to the orthorhombic structure in nanoconfined ammonia borane. The MCM-41 and SBA-15 scaffolds share the same chemical composition (both SiO_2_) but different morphologies in pore size and surface area. Both are hexagonal mesoporous materials with long channels; however, the pore sizes range between 2.0 and 6.5 nm for MCM-41 and between 4.6 and 30 nm for SBA-15. In this study, we report on the effects of these two different scaffolds as nanoconfinement environments on the thermodynamic properties of ammonia borane. Previous research indicates that AB behaviors in MCM-41 depend significantly on the level of AB loading: ammonia borane remains uniformly distributed within the mesopores of MCM-41 when synthesized at an AB to MCM-41 ratio of 1:2 (50 wt% of AB), but aggregates outside the mesopores when AB content exceeds 50 wt.% [37,38,39]. Therefore, in this study, AB/SBA-15 and AB/MCM-41 samples containing 50 wt.% of AB were prepared and analyzed for their low temperature properties.

## 2. Materials and Methods

Ammonia borane powder, with a purity greater than 97%, was purchased from Sigma Aldrich, St. Louis, MO, USA and used without further purification. SBA-15, with a pore size of approximately 8 nm and a Brunauer–Emmett–Teller (BET) surface area of ~650 m^2^/g, was sourced from Novel Chemistry Corp. MCM-41, with a pore size of ~2.1–2.7 nm and a BET surface area of ~1000 m^2^/g, was obtained from Sigma Aldrich. The MCM-41 nanoconfined ammonia borane sample was prepared according to the procedure described in previous studies [37,38,39]. A solution of AB (50 mg) in tetrahydrofuran (THF, 1 mL) was gradually added to MCM-41 (100 mg) in small aliquots, allowing capillary action to drive the solution into the MCM-41 channels. The infiltrated MCM-41 was then dried by vacuum evaporation, producing an MCM-41 sample coated internally with ammonia borane at an approximately 1:2 AB to MCM-41 ratio. The BET surface area of the MCM-41/AB sample was measured to be around 80 m^2^/g, indicating the successful infiltration of ammonia borane into the mesopores. The same procedure was used to synthesize the SBA-15 nanoconfined AB sample.

A symmetric diamond anvil cell (DAC) with two type-I gem-quality diamonds of 400 μm culet size was used for containing the sample during the low-temperature experiment. A stainless steel gasket was prepared with a hole of 250 μm diameter drilled in the center as a sample chamber. Bulk ammonia borane and SBA-15-confined ammonia borane samples were loaded in the sample chamber side by side. A few small ruby spheres were loaded in the sample chamber for monitoring the possible pressure change during the experiments, based on the pressure dependence of ruby fluorescence [40,41]. On the backside of one diamond anvil, a few ruby spheres were also placed for an in situ comparison of the fluorescence from the ruby spheres inside and outside the sample chamber at each temperature point. The cell was loaded in the cryogenic system of liquid nitrogen cooling medium with an optical window access to the sample. The Raman spectra of the sample were collected during cooling with temperature regulated by a Cryogenic Temperature Controller equipped with two Si-diode sensors. We use Raman spectroscopy to investigate the effect of mesopore size on the phase transition of ammonia borane, as Raman shifts are highly sensitive to atomic bonding within molecular crystals. This sensitivity makes Raman spectroscopy an ideal tool for detecting subtle changes in the molecular structure associated with the phase transformation. At each data collection step, the system was held for approximately 15 min to reach thermal equilibrium. Raman spectroscopy was conducted by using a 514 nm Ar^+^ laser as an excitation source. The phase transition from the tetragonal to the orthorhombic structure was identified by observing the slitting of certain characteristic peaks.

## 3. Results

Raman spectra were collected as a function of temperature (room temperature to 90 K) for neat ammonia borane and SBA-15-confined ammonia borane in one run, and for MCM-41-confined ammonia borane in a separate run. When the ammonia borane is confined within the mesoporous silica, the Raman signals weaken compared to those of neat ammonia borane. Additionally, fluorescence from the diamond anvils introduces a high background in the spectra. Raman peak assignments were adopted from a previous study [35] that utilized theoretical modeling through the harmonic analysis of the periodic electronic structure of 16 ammonia borane molecules arranged in 2 × 2 × 2 unit cells. A summary of the Raman peak assignments for the tetragonal and orthorhombic phases observed in this study (neat ammonia borane) and in the previous study (single crystal ammonia borane) is provided in Table 1.

### 3.1. Neat Ammonia Borane

**Figure 1:**
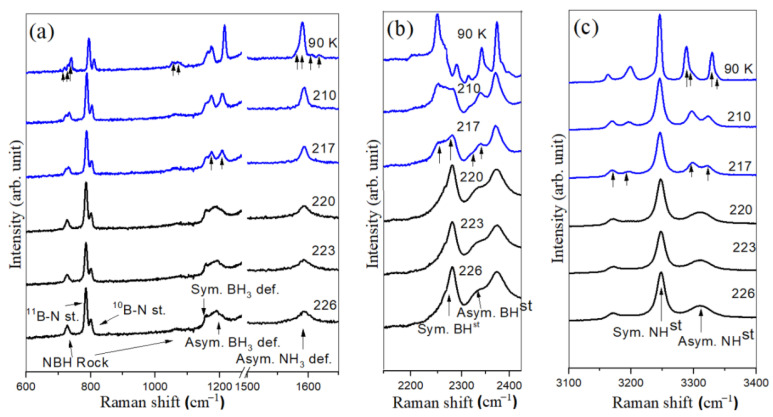
Selected Raman spectra of neat NH_3_BH_3_ at different temperatures in the spectral region of (**a**) 600–1650 cm^−1^, (**b**) 2100–2500 cm^−1^ and (**c**) 3100–3400 cm^−1^. The intense Raman peak of the diamond anvil is truncated in the region of 1270–1500 cm^−1^. The sample temperature is indicated in each spectrum. Arrows indicate peak slitting in the spectra of 217 K and 90 K.

Figure 1 presents selected Raman spectra of neat ammonia borane collected during cooling. The Raman spectra of tetragonal ammonia borane (collected at 226 K) are conveniently classified into several segments based on molecular features: (a) N–H rocking and deformation modes, B–H rocking and deformation modes, and the B–N stretching region (600–1650 cm^−1^), (b) B-H stretching region (2100–2500 cm^−1^), and (c) N–H stretching region (3100–3400 cm^−1^). The observed Raman peaks in these regions align well with those reported in previous studies [28,35,42,43]. As the sample is cooled, the tetragonal to orthorhombic phase transition of bulk ammonia borane occurs around 217 K. The transformation to the orthorhombic phase is characterized by the factor group splitting of Raman modes described by previous studies [35,44]. For example, upon this phase transformation, the asymmetric N-H stretching peak at 3311 cm^−1^ splits into two peaks just below the phase transition temperature and then into four peaks, consistent with full factor group splitting, which is evident only at a much lower temperature. Additionally, the symmetric N-H stretching mode at 3245 cm^−1^ splits into two modes across the phase transition, while the unassigned mode at 3173 cm^−1^ splits into two modes at 3192 and 3165 cm^−1^. The asymmetric B-H stretching mode at 2328 cm^−1^ and the symmetric B-H stretching mode at 2277 cm^−1^ clearly split into two components. According to factor group analysis, the asymmetric NH_3_ deformation mode at 1595 cm^−1^ splits into four components. Nevertheless, the splitting is so subtle that the resolution of the spectrometer (~4 cm^−1^) is insufficient to immediately distinguish it following the transition. At a large temperature overstep, however, the splitting becomes discernible in spectra collected at 90 K. The asymmetric BH_3_ deformation mode splits into two modes. The NBH rocking mode at 1055 cm^−1^ becomes intense during cooling and splits into three modes. The lower frequency rocking mode at 727 cm^−1^ displays a similar behavior and splits into three peaks upon phase transition and are well resolved at the collected spectra at 90 K. ^11^B–N and ^10^B–N stretching modes appear at 800 and 818 cm^−1^, respectively. No peak splitting is observed at tetragonal to orthorhombic phase transition for the BN stretching region.

### 3.2. SBA-15-Confined Ammonia Borane

**Figure 2:**
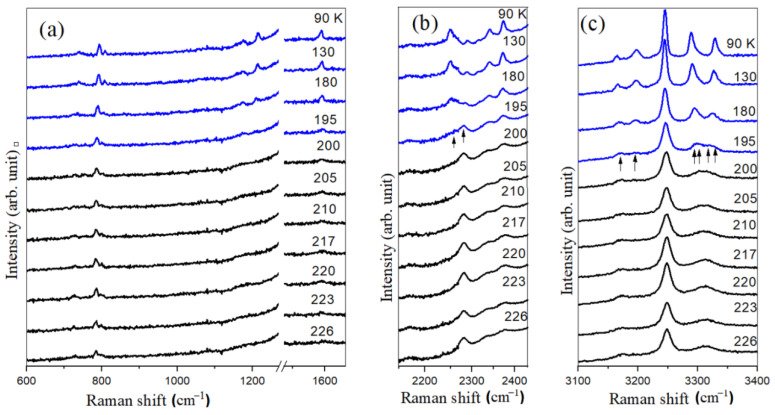
Selected Raman spectra of SBA-15/NH_3_BH_3_ at different temperatures in the spectral region of (**a**) 600–1650 cm^−1^, (**b**) 2100–2500 cm^−1^ and (**c**) 3100–3400 cm^−1^. The intense Raman mode of the diamond anvil is truncated in the region of 1270–1500 cm^−1^. The sample temperature is indicated in each spectrum. Arrows indicate peak slitting in the spectra of 195 K.

Figure 2 shows the collected Raman spectra of SBA-15-confined ammonia borane. No sign of the phase transformation was observed at 217 K as the sample temperature was lowered. The tetragonal to orthorhombic phase transition is depressed down to a lower temperature. The splitting of asymmetric N-H stretching and asymmetric and symmetric B-H stretching are clearly discernible in the spectrum upon cooling down to 195 K. The splitting of asymmetric NH_3_ deformation, BH_3_ deformation and NBH rocking modes are still observed, although poorly resolved. The intensity of these low frequency modes is very low due to the fact that ammonia borane resides inside the pores. The lowering of the phase transformation temperature can be attributed to the nanoconfinement effect of SBA-15. Upon further cooling down to 90 K, we did not observe any other phase transformation.

### 3.3. MCM-41-Confined Ammonia Borane

**Figure 3:**
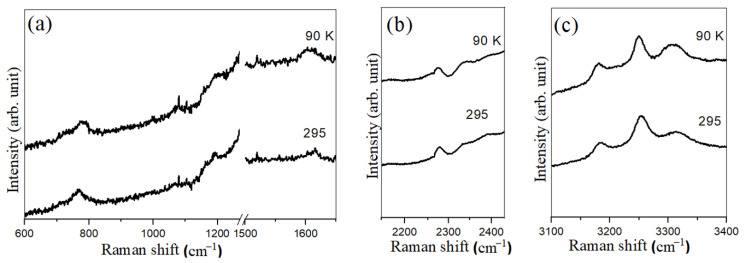
Selected Raman spectra of MCM-41/NH_3_BH_3_ at different temperatures in the spectral region of (**a**) 600–1650 cm^−1^, (**b**) 2100–2500 cm^−1^ and (**c**) 3100–3400 cm^−1^. The intense Raman mode of the diamond anvil is truncated in the region of 1270–1500 cm^−1^. The sample temperature is indicated in each spectrum.

During the cooling of MCM-41-confined ammonia borane from room temperature to 90 K, no characteristic change was observed in the Raman spectra. Figure 3 shows the collected Raman spectra of MCM-41-confined ammonia borane at the beginning and ending temperatures. As ammonia borane is embedded inside the mesoporous silica of an even smaller pore size, the Raman signal becomes weaker and the low frequency Raman peaks are clearly hardly resolved. Still, some modes are discernible, like the N-H and B-H stretching modes. It is clear that no splitting of the N-H and B-H characteristic Raman modes of tetragonal ammonia borane occur during cooling down, indicating that the tetragonal to orthorhombic phase transition may be depressed to a temperature below 90 K. This is consistent with the previous observation by Kim et al. [37] and Paolone et al. [38].

## 4. Discussion

In 1976, Buffat and Borel described the size dependence melting transition in a nanoparticle system [45]. In the case of a solid–liquid phase transition, a reduction in particle size generally leads to a melting point depression. This phenomenon arises from factors contributing to the total energy of a nanocrystal system: in a system consisting of only a few hundred atoms, a substantial fraction of these atoms resides on the surface. Because surface atoms are unsaturated in bonding, a large amount of energy is associated with this surface. Conversely, in the dynamic liquid phase, surface atoms can move to minimize surface area, causing a reduction in the surface energy. Thus, on melting, the liquid phase is stabilized, reducing the surface energy. The smaller the nanocrystal, the larger the contribution made by the surface energy to the overall energy of the system and thus the more dramatic the melting point depression. The relationship between the modification of transition temperature and the crystal size can be expressed as follows [2,45,46]:

$$T_{b}-T_{m}=\frac{2T_{m}}{\Delta H{}^{\circ}\rho_{sol}R_{sol}}\left[\gamma_{sol}-\gamma_{liq}{\left(\frac{\rho_{sol}}{\rho_{liq}}\right)}^{⅔}\right]$$

where *T_b_* and *T_m_* represent the transition (melting) temperatures of bulk and size-modified samples, respectively, *R* is the particle radius, *ΔH°* is the enthalpy change for the phase transition, *ρ* is density and *γ* is the surface tension of each phase.

From the surface energy point of view, a reduction in nanocrystal size may either increase or decrease the solid–solid phase transition temperature depending on the relative surface energy of the phases before and after the transition, or depending on the sign of the term in the parentheses of above equation [2,45,46]. This is also due to the fact that the surface energy becomes more significant in the nanocrystal system. The competition between the surface energy and internal energy of each crystallographic phase is controlled by the crystal size and determines the stable phase. As the surface area to volume ratio increases, i.e., the crystal size decreases, phases with lower surface energy become more favorable. The possible adsorption of the sample on pore walls may also alter the sample surface energy. But its influence on the surface energy is likely much less significant than the grain size effect.

Our study observes that the tetragonal to orthorhombic phase transition temperature decreases with the decreasing pore size of the nanoconfinement. The pore size of the nanoconfinement template effectively controls the grain size of the ammonia borane within it. As the grain size decreases, the tetragonal phase becomes increasingly stable. This stabilization is likely due to the lower surface energy of the tetragonal phase compared to the orthorhombic phase, making the tetragonal phase more favorable as the grain size of the nanoconfined ammonia borane is reduced.

Previously, Gutowska et al. [1] reported a significant improvement in the hydrogen storage properties of ammonia borane nanoconfined inside SBA-15, i.e., the hydrogen released at a lower temperature, there was an increased purity of hydrogen with suppression of the release of toxic byproduct (borazine) and a modification of the enthalpy of the decomposition (theenthalpy of dehydrogenation becomes less exothermic). Later, Lai et al. [30] and Sullivan et al. [31] studied the thermal decomposition behavior of SBA-15 and MCM-41 nanoconfined ammonia borane. They reported controversies regarding the size dependence or size independence of dehydrogenation. In this study, we observed the size dependence of the transition temperature, i.e., a smaller pore size suppresses the phase transition to a lower temperature, indicating that the mesoporous silica scaffolds influence the phase transition and the dehydrogenation through different mechanisms. While the phase transition is dominantly affected by the change in surface energy in the nanoconfinements, the dehydrogenation is more influenced by the chemical interaction between the ammonia borane molecules and silica of the confinement [30,31].

## 5. Conclusions

The in situ Raman spectroscopic study demonstrates the dependence of the solid–solid phase transition temperature of ammonia borane embedded in mesoporous silica on pore size. The temperature-induced tetragonal to orthorhombic phase transition is suppressed from ~217 K in neat ammonia borane to ~195 K in SBA-15 nanoconfined ammonia borane, and further down to below 90 K in MCM-41 nanoconfined ammonia borane. The pore size of the nanoconfinement controls the grain size of ammonia borane within, which in turn affects its characteristic tetragonal (*I4mm*) to orthorhombic (*Pmn2_1_*) phase transition temperature. The observed decrease in the transition temperature with reduced grain size (smaller pore size) can be attributed to the lower surface energy of the tetragonal phase compared to the orthorhombic phase. The nanoconfinement offers a promising strategy for modifying the physical and chemical properties of other bulk hydrogen storage materials, including, but not limited to, structural phase transformation, dehydrogenation and rehydrogenation. A further investigation into the fundamental properties of nanoconfined ammonia borane in mesoporous silica is needed to optimize its physical and chemical behavior for hydrogen storage applications.

## Figures and Tables

**Table 1:** Raman peak assignments of ammonia borane observed under different conditions.

Assignment	Tetragonal Phase (cm^−1^)		Orthorhombic Phase (cm^−1^)	
	This Study (226 K)	Ref. [35] (298 K)	This Study (90 K)	Ref. [35] (88 K)
Asymmetric N-H stretch	3311	3316	3320	3338
			3314	3331
			3300	3300
			3286	3290
Symmetric N-H stretch	3245	3250	3240	3247
			3238	3240
Unassigned	3173	3176	3192	3202
			3165	3165
Asymmetric B-H stretch	2328	2328	2355	2356
			2330	2343
Symmetric B-H stretch	2277	2279	2288	2289
			2270	2263
NH_3_ deformation	1595	1600	1600	1622
			1595	1609
			1590	1593
			1583	1585
BH_3_ deformation	1185	1189	1217	1220
				1214
			1189	1180
				1173
NBH rock	1055	1065	1084	1086
			1065	1073
			1054	1056
B-N stretch	818	800	816	813
				810
B-N stretch	780	784	799	798
				794
NBH rock	727	727	760	740
			740	731
			730	721

## Data Availability

The data presented in this study are available on request from the corresponding author due to privacy.
